# Stimulating medical students interest in research: a neglected craft in Africa

**Published:** 2012-09-14

**Authors:** Abdulrasheed Ibrahim, Malachy Eneye Asuku

**Affiliations:** 1 Department of surgery, Ahmadu Bello University Teaching Hospital, P.M.B 06 Shika Zaria, Nigeria

**Keywords:** Medical student, research, interest, Africa

## Abstract

Research as a component of the undergraduate curriculum has not received the deserved attention in many medical schools in Africa. This correspondence underscores the need to reassess and improve on the existing metrics of research among medical students in Africa.

## To the editors of the Pan African Medical Journal

Research as a component of the undergraduate curriculum has not received the deserved attention in many medical schools in Africa [1–3]. This correspondence underscores the need to reassess and improve on the existing metrics of research among medical students in Africa. Although the highest burden of disease is concentrated in low and middle-income countries (LMICs), data from the Institute for Scientific Information shows profound gaps in research output between industrialized and developing settings [4]. This exemplifies the dire need for a stimulation of research. A viable long-term strategy for promoting health research is to target medical students early in their careers. Most of the research to date on the effectiveness of such a strategy has been done in Western settings [5–6]. This research has shown that encouraging research among medical students in their formative years is an important paradigm shift in stimulating interest in future research. It will provide them with a significant exposure to the ethics, methods, controversies and challenges inherent in research and maintain their interest as their careers progress [7].

### Barriers to research

Against a background of severe shortages in health personnel, weak or unavailable research infrastructure and a desperate lack of funds in most medical schools, participating in research by medical students is an added responsibility, not a graduation requirement [2–3]. Thus most students would rather concentrate on preparing for assessment tests and university examinations. Furthermore Consultants have to tandem research responsibilities with an overwhelming demand for clinical services. Faced with continuing pressure to reduce waiting times anything which affects output in clinical services has to come second. Inevitably, research is put at risk as the consequences are considered less than those for not fulfilling service commitments[1]. Given the very feeble research capacity in Africa, research and clinical medicine should be vertically integrated. Excellent research questions stem from astute clinical observations. The call for a focus on undergraduate research is essentially about developing these observations.

### Potential strategies

We must acknowledge the different priorities of a younger generation and make efforts to bridge this generation gap. Reaching out to our successors must begin with an enthusiastic mentorship of our medical students in research. Students seek and emulate respected role models. Members of Faculty with outstanding track record in research will serve as trusted guides. Such mentoring relationships will ensure that graduating medical students are competent in both patient care and application of evidenced based medicine. Medical schools should establish Student Research Units with a vision of promoting the culture of scholarly publication that are relevant and beneficial to the peculiar health problems in Africa. This unit will also facilitate twinning arrangements between medical schools in the developed countries and African medical schools to foster locally driven collaborative research.

Faculties of Postgraduate Colleges in Africa need to introduce student sections during their conferences, research workshops, and Journal publications. Participation at conferences and workshops will be encouraged by provision of travel scholarships. We need to convey the excitement, triumphs and personal reward we derive from research and publication. Medical students should also have the thrill of seeing their names “in lights” (ie, in print) for the first time as authors of a published article [8].

Quality research is unthinkable without appropriate funding. This begs the question of how it can be boosted as well as its diligent allocation. Telecommunication, pharmaceutical and medical device companies should be made to play a greater role as donor agencies. There support must meet the needs for a feasible and sustainable research capacity among medical students.

## Conclusion

European medical students had to write and defend a research thesis for their doctorate in the latter half of the 19th century. Heparin, insulin, the sinoatrial node, ether anaesthesia and Spermatozoa are some of the major discoveries made on this platform by medical students [9]. It is a given that research in science remains fundamental for the industrialized world. For us in Africa, research and its immense benefits are almost a question of survival.
